# Hepatic Arterial Infusion Chemotherapy with Serplulimab and the Bevacizumab Biosimilar HLX04 for Advanced Hepatocellular Carcinoma: A Prospective, Observational Phase II Clinical Trial

**DOI:** 10.3390/cancers17193235

**Published:** 2025-10-05

**Authors:** Huikai Li, Tongguo Si, Rentao Li, Xiaojing Xie, Yang Liu, Linlin Fu, Yu Bai, Junchao Yao, Xihao Zhang, Mao Yang, Xiaofeng Mu

**Affiliations:** 1 Liver Cancer Center, Tianjin Medical University Cancer Institute & Hospital, National Clinical Research Center for Cancer, Key Laboratory of Cancer Prevention and Therapy, Tianjin’s Clinical Research Center for Cancer, Tianjin 300060, China; yang_liu@tmu.edu.cn; 2 Invasive Interventional Therapy Department, Tianjin Cancer Hospital Airport Hospital, National Clinical Research Center for Cancer, Tianjin 300380, China; sitongguo@tjmuch.com (T.S.); matchym@126.com (M.Y.); 3 Department of Hepatobiliary and Pancreatic Oncology, Tianjin Cancer Hospital Airport Hospital, National Clinical Research Center for Cancer, Tianjin 300308, China; lirentao1990@163.com (R.L.); xxjfjxxjfj@163.com (X.X.); fulinlin2023@163.com (L.F.); yuwhite@163.com (Y.B.); yaojunchao.123@163.com (J.Y.); zxh15510801035@163.com (X.Z.)

**Keywords:** hepatocellular carcinoma, serplulimab, hepatic arterial infusion chemotherapy

## Abstract

Background/Objectives: Advanced hepatocellular carcinoma (HCC) presents limited treatment options; however, immunotherapy demonstrates encouraging outcomes and acceptable adverse reactions in advanced HCC. This study evaluates the efficacy and safety of combining serplulimab, the bevacizumab biosimilar HLX04, and hepatic arterial infusion chemotherapy (HAIC) as a first-line therapy. Methods: This prospective, observational, single-center phase II trial enrolled untreated HCC patients with Barcelona Clinic Liver Cancer (BCLC) stage C. All patients received serplulimab (4.5 mg/kg) and HLX04 (15.0 mg/kg) every 3 weeks, followed by the HAIC-FOLFOX regimen. The primary endpoint was the objective response rate (ORR). Secondary endpoints included the disease control rate (DCR), progression-free survival (PFS), and safety. Results: A total of 32 patients were enrolled. The best outcomes showed an ORR of 53.1%, including 17 partial responses (PR, 53.1%) and 12 stable diseases (SD, 37.5%), resulting in a DCR of 90.6%. Subgroup analysis showed a higher ORR in patients with a single lesion and those receiving ≥3 treatment cycles, with an ORR of 60.7% in the latter group. Additionally, five patients underwent successful hepatectomy after ≥3 treatment cycles, with postoperative pathology confirming extensive tumor necrosis. Kaplan–Meier analysis estimated PFS rates of 89.9% (95% CI: 79.5–100.0%) at 6 months and 70.8% (95% CI: 54.2–92.4%) at 12 months. No deaths related to adverse events (AEs) occurred; four (12.5%) patients experienced grade IV AEs and twelve (37.5%) patients experienced grade III AEs. Conclusions: Serplulimab, HLX04, and HAIC combined as a first-line treatment for advanced HCC have demonstrated promising efficacy, particularly in patients completing ≥3 cycles, with an acceptable safety profile. Further investigation in larger trials is required.

## Simple Summary

This prospective phase II study evaluated the efficacy and safety of combining serplulimab, the bevacizumab biosimilar HLX04, and hepatic arterial infusion chemotherapy (HAIC) as a first-line treatment in patients with advanced hepatocellular carcinoma (HCC). Among the 32 enrolled patients, the combination therapy achieved a promising objective response rate (ORR) of 53.1% and a disease control rate (DCR) of 90.6%, with favorable progression-free survival at 6 and 12 months. The regimen showed acceptable tolerability. Four (12.5%) patients experienced grade IV adverse events (AEs) and twelve (37.5%) experienced grade III AEs, with no toxic death owing to AEs. These results support further investigation of this combined approach in larger clinical trials.

## 1. Introduction

Primary liver cancer is one of the most common and most malignant tumors in the world [1]. According to the Global Burden of Disease (GBD) 2021 estimates, 866,136 new liver cancer cases and 758,725 liver cancer deaths occurred, with the mortality rate ranking third and the incidence rate ranking sixth globally [2]. Notably, China contributed to nearly 42.0% of the global cases and deaths (367,657 and 316,544, respectively), positioning liver cancer as the fourth most frequently diagnosed malignancy and the second leading cause of cancer-related mortality in the nation [2,3].

Previous research has confirmed that chemotherapy drugs provide local disease control and significant survival advantages for individuals with advanced hepatocellular carcinoma (HCC), and it has been demonstrated to be both safe and effective [4,5]. At present, oxaliplatin is approved in China for the management of locally advanced or metastatic HCC that cannot be treated through surgery or localized therapies [6,7,8]. Findings from a phase III clinical trial have demonstrated that hepatic arterial infusion chemotherapy (HAIC) using the FOLFOX regimen (HAIC-FOLFOX) markedly extends overall survival (OS) in patients with large tumors and offers a more favorable safety profile compared to transarterial chemoembolization (TACE) [9]. The survival outcomes of HCC patients were greatly increased by combining HAIC-FOLFOX with targeted sorafenib compared to using sorafenib alone [10]. Meanwhile, studies on PD-1 inhibitors have confirmed their remarkable benefits for patients with advanced HCC [11]. Additionally, vascular endothelial growth factor (VEGF) has been implicated as a key factor in facilitating tumor immune escape, with preclinical and clinical data suggesting that VEGF exerts immune-suppressive effects on HCC [12]. As a result, immune checkpoint inhibitors (ICIs) are being incorporated into HCC treatment, while combining molecularly targeted therapies with immunotherapy is increasingly recognized as a promising approach to strengthen the immune response against this cancer [13]. The phase III IMbrave 150 study indicated clinically meaningful benefits of combining the PD-L1 inhibitor (atezolizumab) and a VEGF antibody (bevacizumab) for unresectable HCC [14]. ICIs, alongside combined antiangiogenic agents, have transformed the therapeutic approach for advanced HCC in clinical settings. Currently, more exploratory investigations have indicated that combining chemotherapy with targeted drugs and ICIs is feasible and more beneficial for the treatment of HCC [15,16,17]. 

Serplulimab, a PD-1 inhibitor with the broadest approved indications, is authorized for treating non-small-cell lung cancer (NSCLC), small-cell lung cancer (SCLC), and esophageal squamous-cell carcinoma (ESCC) [18]. The ASTRUM-004 trials demonstrated that incorporating serplulimab into chemotherapy significantly extended progression-free survival (PFS) compared to a placebo and chemotherapy in previously untreated patients with locally advanced or metastatic squamous NSCLC, underscoring its potential as a first-line treatment option for lung cancer. HLX04, the bevacizumab biosimilar, demonstrated in a phase 1 randomized study that it had similar safety and pharmacokinetic profiles to reference bevacizumab in healthy Chinese males, and a phase III study further investigated the efficacy and safety equivalence between HLX04 and bevacizumab in patients with metastatic colorectal cancer (NCT03511963) [19,20]. Additionally, two phase II clinical studies on serplulimab showed that combining it with HLX04 achieved good clinical efficacy in treating HCC [21,22]. This combination has exhibited a manageable safety profile alongside preliminary efficacy when used as a first-line treatment for advanced HCC patients [21,22].

Immunotherapy combined with anti-VEGF or chemotherapy has shown promise in several cancers, potentially reducing resistance and improving outcomes [23,24,25]. However, clinical evidence of a synergism between serplulimab, HLX04, and local chemotherapy in advanced HCC is largely lacking. To address this gap, we conducted a prospective phase II trial to evaluate the effectiveness and safety of serplulimab combined with HLX04 and HAIC as a first-line treatment for advanced HCC.

## 2. Materials and Methods

### 2.1. Study Design

This single-center, observational, phase II prospective study enrolled patients with untreated HCC at Barcelona Clinic Liver Cancer (BCLC) stage C who agreed to receive HAIC plus serplulimab and HLX04 as a first-line therapy. Recruitment took place between August 2023 and September 2024 at Tianjin Cancer Hospital Airport Hospital, with the study duration lasting for 2 years (from 15 February 2023, to 15 February 2025). The minimum follow-up was 6 months, unless the patient was deceased, the disease progressed, or the patient was lost to follow-up. Informed consent was obtained from participants regarding their participation and data storage and handling. The study was reviewed and approved by the Ethics Committee of Tianjin Cancer Hospital Airport Hospital (approval no. EC-2023-0032) and was registered on ClinicalTrials.gov under registration number NCT06370065.

### 2.2. Patients

Inclusion criteria included the following: (i) age ≥ 18 years; (ii) diagnosed with HCC; (iii) no prior systemic therapy for HCC; (iv) at least one measurable target lesion based on RECIST (Response Evaluation Criteria in Solid Tumors) v1.1; (v) Child–Pugh Grade A or well-compensated Grade B (≤7 points); (vi) ECOG (Eastern Cooperative Oncology Group performance status) performance status of 0–1; and (vii) life expectancy ≥12 weeks.

Exclusion criteria included the following: (i) known intrahepatic cholangiocarcinoma, mixed cellular carcinoma, or fibrolamellar carcinoma; (ii) other active malignancies within the past 5 years; (iii) prior organ or allogeneic bone marrow transplantation; (iv) uncontrolled pleural or pericardial effusion or a condition requiring frequent ascites drainage; (v) history of gastrointestinal bleeding, abdominal fistula, gastrointestinal perforation, or abdominal abscess within the past 6 months; (vi) confirmed central nervous system or leptomeningeal metastases; and (vii) heart failure NYHA (New York Heart Association) > II or LVEF (left ventricular ejection fraction) < 50% on an echocardiogram. More detailed inclusion and exclusion criteria are provided in Appendix A.

### 2.3. Treatment

Patients were given intravenous serplulimab (4.5 mg/kg, Shanghai Henlius Biopharmaceutical Co., Ltd., Shanghai, China) and HLX04 (15.0 mg/kg, Shanghai Henlius Biopharmaceutical Co., Ltd., Shanghai, China), along with FOLFOX chemotherapy for HAIC, every 3 weeks per treatment cycle. The dosage of serplulimab was determined based on its application dose and safety of the previous phase I and II studies, according to the researcher’s research plan, and the dosage of HLX04 was based on Roche’s IMbrave150 study, in which the dose of bevacizumab was 15 mg/kg/3 w [26]. Participants received FOLFOX-regimen chemotherapy through the hepatic artery. Day 1 of each cycle involved 85 mg/m^2^ of oxaliplatin (2 h via hepatic artery infusion) and 400 mg/m^2^ of calcium folinate (LV) (2 h via intravenous infusion), followed by 400 mg/m^2^ of fluorouracil (via hepatic artery infusion) and 2400 mg/m^2^ of fluorouracil (44–46 h via hepatic artery continuous infusion). In the first cycle, serplulimab was administered on Day 1, followed by HLX04 on Day 2. If an infusion reaction occurred during the initial administration, this schedule was maintained for subsequent cycles. Otherwise, both drugs were administered during the same treatment session in later cycles, with a minimum interval of 30 min and no more than 1 day between infusions. HAIC treatment (D1–3) started at least 1 h after infusion completion. 

HAIC therapy was administered for a maximum of 8 cycles, while serplulimab and HLX04 were continued for up to 2 years or until the occurrence of any of the following events, whichever came first: mortality, disease progression, no clinical benefit, informed consent withdrawal, pregnancy, or the development of intolerable toxicity. 

### 2.4. Study Endpoints and Assessments

The primary endpoint was the objective response rate (ORR), defined as the proportion of patients achieving complete response (CR) or partial response (PR). Secondary endpoints included the disease control rate (DCR), defined as the proportion of patients achieving CR, PR, or stable disease (SD); progression-free survival (PFS), defined as the time from the start of trial treatment until progressive disease (PD) or death; and safety. Study treatment visits were conducted every 3 weeks (±3 days). Adverse events (AEs) were categorized using the Medical Dictionary for Regulatory Activities and graded according to the National Cancer Institute’s Common Terminology Criteria for Adverse Events, version 5.0.

The investigators and the Independent Radiology Review Committee evaluated tumor imaging using RECIST v1.1 criteria. Radiological examinations were conducted every 9 weeks (±7 days) within the first 96 weeks, then every 12 weeks (±7 days) afterward. If PD was initially observed, a confirmatory imaging assessment was required at the next tumor evaluation (at least 4 weeks later) to verify progression. Treatment was discontinued upon confirming a second PD.

### 2.5. Statistical Analysis

Sample size estimation: Assuming an ORR of 60% for the target value, with type I error set to α = 0.05 (bilateral) and type II error to β = 0.2, patients must be included in the sample size. To account for censoring and dropout, the plan was to enroll 32 patients with advanced HCC.

All statistical analyses were performed using SPSS (V.24; IBM, Armonk, New York, NY, USA) and Prism (V.8; GraphPad Software, San Diego, CA, USA). The baseline characteristics of the patients with advanced HCC were described in detail, including the rates of HBV infection, portal vein thrombosis, extrahepatic metastasis, and clinical staging. The ORR and DCR were estimated based on treatment outcomes, and the effects on operable patients were investigated. The Kaplan–Meier product limit method was also used to calculate the cumulative PFS rate, and the Cox proportional hazards model was used to estimate hazard ratios (HRs).

## 3. Results

### 3.1. Patient Characteristics 

In the final analysis, 32 patients were included from a total of 35 screened, of whom 3 were excluded due to loss to follow-up without efficacy data (Supplementary Figure S1). The median age was 62 years (range: 53.0~66.8), and the sample comprised 26 (81.2%) men and 6 (18.8%) women. The prevalence of HBV infection was high at 68.8%, with significant proportions of patients having cirrhosis and portal vein thrombosis, at 62.5% and 50.0%, respectively. According to the China Liver Cancer Staging (CNLC) system, two (6.3%) patients were classified as stage Ib, two (6.3%) as stage IIa, five (15.6%) as stage IIb, fourteen (43.8%) as stage IIIa, and nine (28.1%) as stage IIIb. Based on the American Joint Committee on Cancer (AJCC) staging system, one (3.1%) patient was classified as stage II, twenty-four (75.0%) as stage III, and seven (21.9%) as stage IV. As of the data cutoff, 28 (87.5%) patients had completed at least three treatment cycles (Table 1). 

### 3.2. Efficacy

**Figure 1:**
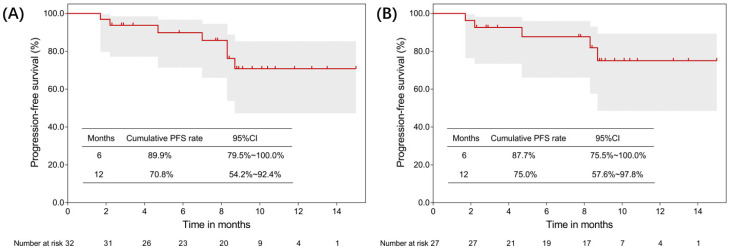
Kaplan–Meier curves for progression-free survival (PFS). (**A**) PFS for all patients (n = 32); (**B**) PFS after excluding patients who underwent partial hepatectomy (n = 27).

**Figure 2:**
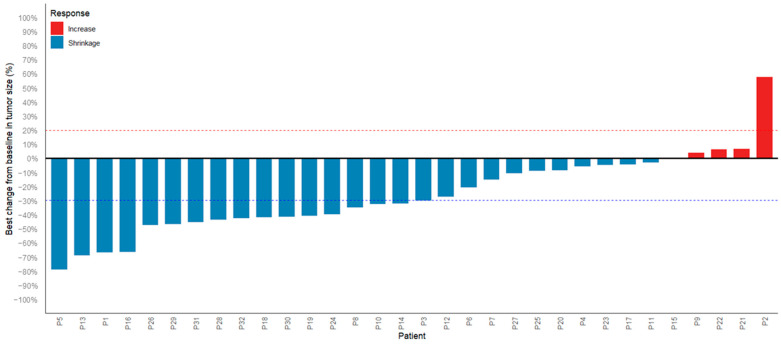
Waterfall Plot of Tumor Size Change (n = 32). Dashed lines indicate RECIST 1.1 thresholds: ≥−30% = Partial Response (Treatment Effective); −30% to +20% = Stable Disease; ≥+20% = Progressive Disease.

As of the cut-off date for analysis (December 2024), 32 patients were evaluable for treatment response. The best responses were as follows: 17 patients achieved PR (53.1%), 12 had SD (37.5%), and 3 had PD (9.4%), resulting in an ORR of 53.1% (95%CI, 34.7–70.9) and a DCR of 90.6% (95%CI, 75.0–97.5) (Table 2). Three patients had PD from the start, two progressed from PR to PD, and two deteriorated from SD to PD. A total of 28 patients completed three or more cycles of treatment, with an ORR of 60.7%. Five patients with stage IB to IIIA liver cancer underwent partial hepatectomy after three or four cycles of treatment, with four confirmed PR and one SD (Table 3). Survival analysis showed that Kaplan–Meier estimates of PFS were 89.9% (95% CI, 79.5–100.0%) at 6 months and 70.8% (95% CI, 54.2–92.4%) at 12 months (Figure 1A). After excluding five patients who underwent partial hepatectomy, PFS was 87.7% (95% CI, 75.5–100.0%) at 6 months and 75.0% (95% CI, 57.6–97.8%) at 12 months (Figure 1B). Furthermore, in the univariate Cox proportional hazards regression analysis for PFS (Supplementary Table S2), none of the evaluated variables reached statistical significance. The waterfall plot provides a detailed display of the changes in tumor lesions before and after treatment for all subjects (Figure 2). 

Subgroup analysis showed that the number of lesions and treatment cycles significantly influenced the ORR. Patients with a single lesion had a higher ORR than those with multiple lesions (100% vs. 44.4%, *p* < 0.05), and those receiving ≥3 cycles of serplulimab/bevacizumab plus TACE/HAIC had a higher ORR than those with <3 cycles (60.7% vs. 0%, *p* < 0.05). Other demographics, comorbidities, and tumor stages were not significantly associated with the ORR (Supplementary Table S1).

### 3.3. Safety

**Figure 3:**
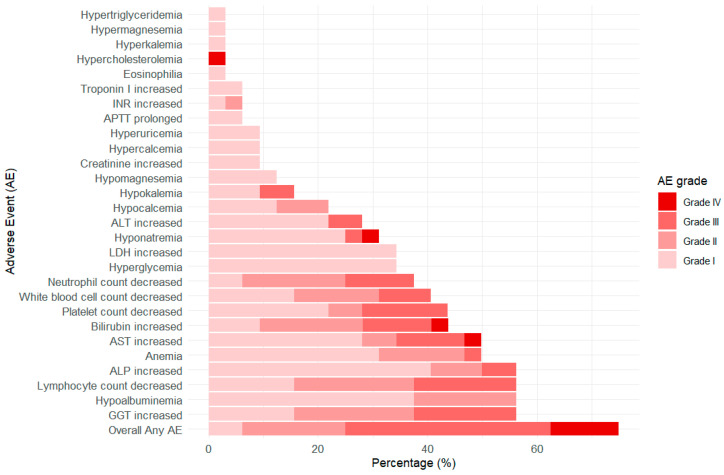
Adverse Events of the Study by Severity Grade (n = 32).

The safety analysis includes all of the enrolled participants. As of December 2024, AEs of any grade (grade I to IV) had occurred in 75% of patients, with grade III or higher AEs recorded in 16 patients (50.0%). The most prevalent grade III or higher AEs included decreased lymphocyte count (18.8%), elevated γ-glutamyltransferase (18.8%), increased aspartate aminotransferase (15.6%), thrombocytopenia (15.6%), elevated blood bilirubin (15.6%), and decreased neutrophil count (12.5%). The majority of AEs were related to abnormal liver function and myelosuppression (Figure 3 and Supplementary Table S3). Among the 32 patients analyzed in detail, 2 of them experienced an AE after serplulimab and HLX04 infusion, resulting in the suspension of TACE/HAIC treatment. There were no other discontinuation events caused by AEs.

## 4. Discussion

This prospective and observational phase II clinical research analyzed the efficacy and safety of HAIC in combination with serplulimab and HLX04, with the best ORR reaching 53.1%. Furthermore, among patients who underwent at least three cycles of therapy, the ORR was 60.7%. The study discovered that chemotherapy, immunotherapy, and combined targeted treatment were safe, with minimal serious side effects.

In China, related studies have shown that approximately 40% of HCC cases diagnosed in China are in the locally advanced stage (CNLC, Chinese Liver Cancer Stage IIb/IIIa). Moreover, it is not recommended for surgical resection according to the guidelines from the American Association for the Study of Liver Diseases and the European Association for the Study of the Liver [27,28,29,30,31]. In this study, after 3–4 cycles of treatment, five patients with PR or SD underwent partial hepatic resection. These data indicate that HAIC coupled with serplulimab and HLX04 may promote the downstaging of advanced HCC, making surgical intervention feasible for some patients and offering them renewed hope.

ICIs and combined anti-angiogenic medicines have changed the therapeutic strategy for advanced HCC [32,33,34]. Preclinical studies have revealed the synergistic therapeutic benefits of immunotherapy in combination with anti-angiogenesis treatment by regulating multiple signaling pathways [35,36,37,38]. Two phase II studies confirmed the efficacy of serplulimab in treating advanced HCC when combined with HLX04 [22,23]. The research discovered that the ORR was 29.3% (95% CI: 18.1–42.7), the mPFS was 7.3 months (95% CI: 2.8–11.0), and the mOS was 20.4 months (95% CI: 15.0–NE) [22]. A phase III clinical trial of a combination therapy with a PD-1 inhibitor, anti-VEGF medication, and non-small-cell lung cancer chemotherapy demonstrated a significant improvement in PFS among patients, which brings new hope for patients with cancer [39]. One clinical trial (NCT04191889) assessed the benefits of HAIC-FOLFOX with camrelizumab and apatinib for HCC. The combination showed promising results with manageable safety concerns [40]. The study comprised 35 patients, with an ORR of 77.1%, a DCR of 97.1%, and a median PFS of 10.38 months. Other trials have shown that HAIC with anti-PD-1 and targeted treatments can extend life, raise the ORR and DCR, and improve the prognosis of HCC patients [41,42,43,44].

A meta-analysis suggested that HAIC coupled with anti-PD-1/anti-PD-L1 (triple treatment) may increase the frequency and seriousness of AEs. However, it resulted in a larger ORR and better PFS and OS than anti-PD-1/anti-PD-L1 plus angiogenesis inhibitors [45]. In our study, the best ORR of 53.1% was higher than that demonstrated in the phase II study of serplulimab and HLX04. The most prevalent AEs were raised transaminase levels, hyperbilirubinemia, and decreased lymphocyte count, which is consistent with findings from previous phase II clinical trials associated with serplulimab and HLX04 [22]. Compared to the phase 2 study of serplulimab with the bevacizumab biosimilar HLX04 [22], we observed a higher incidence of hypoalbuminemia (56.2% vs. 23.0%) and elevated γ-glutamyltransferase (56.2% vs. 9.8%). No cases of asthma, proteinuria, or bleeding were observed. This variability in AEs may reflect individual differences in treatment response. Based on these findings and the results of previous studies, we believe that our clinical trial provides strong evidence supporting the broader application of HAIC-FOLFOX in combination with serplulimab and HLX04. However, comparative studies are still needed in advanced HCC patients to elucidate the differences between chemotherapy alone and the combination of anti-PD-1 and anti-VEGF treatments. Moreover, the mechanisms need to be clarified and the therapeutic strategies optimized. PD-L1 expression in tumor samples from the enrolled patients was not assessed in this study, which may limit our ability to evaluate the relationship between PD-L1 and treatment response. However, previous multiple clinical trials have shown that the correlation between PD-L1 expression and the efficacy of PD-1/PD-L1 inhibitors is inconsistent [14,46]. Due to the relatively short follow-up period in this study, the current data may not fully reflect the long-term mortality risk of HCC patients. We plan to extend the follow-up to collect data on long-term survival and mortality and will report the results in future studies. The study’s limitations include the lack of a comparison, a small sample size, and a relatively short follow-up period. Overcoming these limitations may lead to better research outcomes in similar studies. Therefore, serplulimab, HLX04, and HAIC-FOLFOX combined as a first-line therapy hold promise for future phase III clinical trials with larger sample sizes in advanced HCC. In short, our trial reveals that combined serplulimab, HLX04, and HAIC-FOLFOX offer a potentially effective first-line therapy for advanced HCC with manageable toxicity. 

## 5. Conclusions

Our trial reveals that the combination of serplulimab, HLX04, and HAIC-FOLFOX offers a potentially effective first-line therapy for advanced HCC, showing promising efficacy and acceptable safety. The therapeutic effect was more pronounced in patients who received ≥3 treatment cycles. The therapy also demonstrated potential for tumor downstaging, enabling surgical intervention in some patients. These findings call for further investigation into larger, randomized trials to confirm the benefits of this therapy and optimize treatment strategies for advanced HCC.

## Figures and Tables

**Table 1:** Demographic and baseline characteristics.

Characteristics	Patients (*N* = 32)
Age (year), median (IQR)	62.0 (53.0~66.8)
Sex *n* (%)	
Male	26 (81.2)
Female	6 (18.8)
Drinking history *n* (%)	
No	18 (56.2)
Yes	14 (43.8)
Smoking history *n* (%)	
No	15 (46.9)
Yes	17 (53.1)
Family history of liver cancer *n* (%)	
No	29 (90.6)
Yes	3 (9.4)
HBV infection *n* (%)	
No	10 (31.2)
Yes	22 (68.8)
HCV infection *n* (%)	
No	30 (93.8)
Yes	2 (6.2)
Hypertension *n* (%)	
No	22 (68.8)
Yes	10 (31.2)
Diabetes *n* (%)	
No	25 (78.1)
Yes	7 (21.9)
Cardiovascular and cerebrovascular diseases *n* (%)	
No	29 (90.6)
Yes	3 (9.4)
Cirrhosis *n* (%)	
No	12 (37.5)
Yes	20 (62.5)
Portal vein cancer thrombus *n* (%)	
No	16 (50.0)
Yes	16 (50.0)
Number of lesions *n* (%)	
Single lesion	5 (15.6)
Multiple lesions	27 (84.4)
Lymph node metastasis *n* (%)	
No	29 (90.6)
Yes	3 (9.4)
Extrahepatic metastasis *n* (%)	
No	27 (84.4)
Yes	5 (15.6)
CNLC stage *n* (%)	
Ib	2 (6.3)
IIa	2 (6.3)
IIb	5 (15.6)
IIIa	14 (43.8)
IIIb	9 (28.1)
AJCC stage *n* (%)	
II	1 (3.1)
IIIA	8 (25.0)
IIIB	16 (50.0)
IVA	2 (6.3)
IVB	5 (15.6)
Serplulimab/Bevacizumab + TACE/HAIC cycles *n* (%)	
≥3 cycles	28 (87.5)
<3 cycles	4 (12.5)

n: number; CNLC: China Liver Cancer Staging System; AJCC: American Joint Committee on Cancer; HBV: Hepatitis B Virus; HCV: Hepatitis C Virus; TACE: Transarterial Chemoembolization; HAIC: Hepatic Arterial Infusion Chemotherapy.

**Table 2:** The clinical results of patients after HAIC combination therapy with serplulimab and the HLX04 (Best treatment response).

Indicator	*N* = 32
Number of assessable cases *n* (%)	32 (100)
CR *n* (%)	0 (0.0)
PR *n* (%)	17(53.1) ^1^
SD *n* (%)	12 (37.5)
PD *n* (%)	3 (9.4) ^2^
ORR	17 (53.1, 95%CI: 34.7–70.9)
DCR	29 (90.6, 95%CI: 75.0–97.5)

^1^ Out of 32 assessable patients, a total of 7 developed PD, of which 3 cases progressed from the beginning, and 2 cases were initially assessed as SD and subsequently progressed to PD. The other two cases were initially assessed as PR, and during follow-up, the disease deteriorated to PD. ^2^ 28 patients experienced at least 3 treatment cycles of serplulimab/HLX04+TACE/HAIC, including 17 ORR patients, with an ORR of 60.7% (17/28).

**Table 3:** Surgical patient information.

No.	Age	Gender	HBV Infection	Portal Vein Tumor Thrombus	CNLC Stage	Treatment Cycles (Preoperative)	Preoperative Tumor Assessment	Surgical Method
1	57	Male	Yes	No	Ib	4	SD	Hepatic segments IV, V, VII resection + Cholecystectomy
2	72	Female	Yes	No	IIb	3	PR	Left hepatectomy + Cholecystectomy
3	66	Male	No	Yes	IIIa	3	PR	Right hepatectomy + Right caudate lobectomy + Cholecystectomy
4	50	Male	No	Yes	IIIa	3	PR	Hepatectomy
5	54	Male	Yes	Yes	IIIa	4	PR	Right hepatectomy + Cholecystectomy + Lymph node dissection

HBV: Hepatitis B Virus; CNLC: China Liver Cancer staging system; PR: Partial Response; SD: Stable Disease.

## Data Availability

The data presented in this study are available on request from the corresponding author. The data are not publicly available due to privacy restrictions.

## Supplementary Materials

The following supporting information can be downloaded at: https://www.mdpi.com/article/10.3390/cancers17193235/s1, Figure S1: Flow chart of patient enrollment and exclusion; Table S1: Sub-analysis of ORR according to the different demographics and baseline characteristics; Table S2: Univariate Cox proportional hazards regression analysis for PFS; Table S3: AEs of the study.

## Appendix A. Main Inclusion and Exclusion Criteria

Inclusion Criteria

1. Willing to participate in the clinical study; fully understands the study, has signed the informed consent form (ICF), and is willing and able to complete all trial procedures.
2. Age ≥ 18 years at the time of signing the ICF.
3. Diagnosed with hepatocellular carcinoma (HCC) via histopathology or cytology, or clinically diagnosed based on the American Association for the Study of Liver Diseases (AASLD) criteria.
4. Barcelona Clinic Liver Cancer (BCLC) stage C, with or without portal vein tumor thrombus (VP3–4 or Cheng’s classification II–IV) confirmed by imaging.
5. No prior systemic therapy for HCC, including chemotherapy, sorafenib, regorafenib, lenvatinib, or other small-molecule anti-angiogenic agents.
6. At least one measurable target lesion per RECIST v1.1, which has not previously undergone surgery, radiotherapy, or other local therapies (e.g., radiofrequency ablation, percutaneous ethanol or acetic acid injection, cryotherapy, high-intensity focused ultrasound, transarterial chemoembolization, or transarterial embolization). Previously treated lesions may be selected as target lesions if they show clear progression per RECIST v1.1. Local treatments must have been completed at least 4 weeks before baseline screening, with treatment-related adverse events (AEs) recovered to NCI-CTCAE Grade ≤1.
7. Child-Pugh liver function classification: Grade A or well-compensated Grade B (≤7 points) within 7 days prior to screening.
8. Eastern Cooperative Oncology Group (ECOG) performance status (PS) of 0 or 1 within 7 days prior to screening.
9. Expected survival ≥12 weeks.
10. For patients with HBsAg (+) or HBcAb (+), HBV-DNA must be <2000 IU/mL to be eligible. Patients negative for HCV antibodies or HCV-RNA are eligible. If HCV-RNA is positive, the patient must agree to receive standard antiviral therapy per local guidelines and have ALT/AST ≤ 3 × ULN.
11. Normal major organ function as assessed within 14 days prior to randomization, without prior transfusion, albumin, recombinant human thrombopoietin, or colony-stimulating factor treatment.

**Table A1:** Organ Function Requirements Within 14 Days Prior to Randomization.

System	Parameter	Criteria
Hematology	White Blood Cells (WBC)	≥3.0 × 10^9^/L
	Neutrophils (ANC)	≥1.5 × 10^9^/L
	Platelets (PLT)	≥75 × 10^9^/L
	Hemoglobin (Hb)	≥90 g/L
Liver Function	Total Bilirubin (TBIL)	≤1.5 × Upper Limit of Normal (ULN)
	Alanine Aminotransferase (ALT)	≤5 × ULN (except for patients with HCV-RNA positivity)
	Aspartate Aminotransferase (AST)	≤5 × ULN (except for patients with HCV-RNA positivity)
	Albumin	≥28 g/L
Renal Function/Urinalysis	Creatinine (Cr)	≤1.5 × ULN; if >1.5 × ULN, creatinine clearance rate should be ≥50 mL/min (calculated according to the Cockcroft-Gault formula)
	Proteinuria	Routine urine protein test ≤1+ or quantitative <1.0 g/24 h
Coagulation Function	Prothrombin Time (PT)	≤1.5 × ULN
	Activated Partial Thromboplastin Time (APTT)	≤1.5 × ULN
	International Normalized Ratio (INR)	≤1.5 × ULN

12. Female patients must meet one of the following criteria:
  (1) Postmenopausal (no menstruation for ≥1 year without another known cause)
  (2) Surgically sterile (bilateral oophorectomy and/or hysterectomy)
  (3) If of childbearing potential:
    - Negative serum pregnancy test within 7 days before randomization.
    - Agreement to use highly effective contraception (failure rate <1% per year) or remain abstinent from the time of consent to at least 150 days after the last dose. Highly effective contraception includes bilateral tubal ligation, vasectomy of male partner, hormonal contraceptives that inhibit ovulation, or intrauterine devices (IUDs). Breastfeeding is not allowed.
13. Male patients must agree to abstain or use contraception as follows: If the partner is of childbearing potential or pregnant, the patient must use a condom or remain abstinent from the time of consent to at least 150 days after the last dose. The reliability of abstinence should be assessed based on trial duration, patient preference, and lifestyle. Periodic abstinence (calendar, ovulation, basal body temperature, or post-ovulation methods) and withdrawal are not considered reliable contraceptive methods.

Exclusion Criteria

1. Known intrahepatic cholangiocarcinoma, mixed-cell carcinoma, or fibrolamellar carcinoma.
2. History of hepatic encephalopathy.
3. Portal hypertension with upper gastrointestinal bleeding within 6 months, or esophageal/gastric varices with red signs (gastroscopy required within 6 months before randomization), or high bleeding risk per investigator assessment.
4. Known central nervous system (CNS) or leptomeningeal metastases.
5. Presence of distant metastases (hepatic portal lymph node metastases are permitted).
6. Co-infection with HBV and HCV (detectable HBV-DNA or HBsAg-positive with anti-HCV antibody-positive or detectable HCV-RNA).
7. Other active malignancies within the past 5 years (except cured localized cancers such as basal cell carcinoma, squamous cell carcinoma of the skin, superficial bladder cancer, carcinoma in situ of the prostate, cervix, or breast).
8. Prior or planned organ or bone marrow transplantation.
9. Uncontrolled pleural effusion, pericardial effusion, or ascites requiring frequent drainage (≥ once per month).
10. Stroke, myocardial infarction, unstable angina, or uncontrolled arrhythmia within the past 6 months (including QTc interval ≥450 ms in males or ≥470 ms in females, calculated using the Fridericia formula).
11. Heart failure NYHA > II or LVEF < 50% on echocardiogram.
12. Uncontrolled hypertension despite optimal medical management (systolic BP ≥ 150 mmHg or diastolic BP ≥ 100 mmHg).
13. History of hypertensive crisis or hypertensive encephalopathy.
14. Active HIV infection, tuberculosis, or other active infections.
15. History or current evidence of pulmonary fibrosis, interstitial lung disease, pneumoconiosis, radiation pneumonitis, drug-related pneumonitis, or severely impaired lung function that may interfere with drug safety evaluation.
16. Active or suspected autoimmune disease, except controlled hypothyroidism on replacement therapy or controlled Type 1 diabetes on insulin therapy.
17. Receipt of a live vaccine within 28 days.
18. Systemic corticosteroid (>10 mg/day prednisone or equivalent) or immunosuppressive therapy within 14 days before screening, unless for acute, short-term use (e.g., contrast allergy prophylaxis). Inhaled or topical steroids are permitted.
19. Major surgery within 28 days (defined as requiring ≥3 weeks for recovery before study treatment initiation).
20. Prior treatment with T-cell co-stimulation or immune checkpoint inhibitors, including but not limited to CTLA-4, PD-1, or PD-L1/2 inhibitors.
21. Prior treatment with bevacizumab or its biosimilars.
22. Participation in another clinical trial within 14 days before randomization.
23. Known allergy to monoclonal antibodies, study drug excipients, platinum, or fluoropyrimidine-based chemotherapy agents.
24. Pregnant or breastfeeding women.
25. Evidence of non-gastrointestinal bleeding (e.g., hemoptysis, abnormal vaginal bleeding) at screening or Grade 2 bleeding within 3 months, or Grade ≥3 bleeding within 6 months before consent.
26. Full-dose anticoagulation or thrombolytic therapy within 7 days prior to randomization (except prophylactic anticoagulation for an open intravenous line, provided INR ≤ 1.5 × ULN and APTT ≤ 1.5 × ULN within 14 days before randomization). Prophylactic low-molecular-weight heparin (e.g., enoxaparin 40 mg/day) is allowed.
27. Chronic daily NSAID use. Occasional NSAID use for symptom relief (e.g., headache, fever) is allowed.
28. The following conditions within 6 months:
  (1) Esophagotracheal fistula, gastrointestinal perforation, intra-abdominal fistula, or intra-abdominal abscess.
  (2) Intestinal obstruction or symptoms requiring parenteral hydration/nutrition or enteral feeding.
  (3) Intra-abdominal inflammatory processes, including peptic ulcer, diverticulitis, or colitis.
  (4) Major vascular diseases (e.g., aortic aneurysm requiring repair or recent peripheral arterial thrombosis).
29. Unhealed wounds, active ulcers, or untreated fractures.
30. History of substance abuse or drug dependence.
31. Any other condition deemed unsuitable by the investigator.
