# Supplementary material for: Hepatic Arterial Infusion Chemotherapy with Serplulimab and the Bevacizumab Biosimilar HLX04 for Advanced Hepatocellular Carcinoma: A Prospective, Observational Phase II Clinical Trial
Source: Cancers (Basel). 2025 Oct 5;17(19):3235. doi: 10.3390/cancers17193235 (PMC12523560; doi:10.3390/cancers17193235)
Supplement: Supplementary file 1 [file cancers-17-03235-s001.zip › Supplementary Table S1.pdf]

Supplementary Table S1. Sub-analysis of ORR according to the different demographics and baseline characteristics

Index	ORR		*P* value*
	No	Yes	
Age *n* (%)			0.153
≤60 years	9 (64.3)	5 (35.7)	
>60 years	6 (33.3)	12 (66.7)	
Sex *n* (%)			0.999
Male	12 (46.2)	14 (53.8)	
Female	3 (50.0)	3 (50.0)	
Drinking history *n* (%)			0.999
No	8 (44.4)	10 (55.6)	
Yes	7 (50.0)	7 (50.0)	
Smoking history *n* (%)			0.999
No	7 (46.7)	8 (53.3)	
Yes	8 (47.1)	9 (52.9)	
Family history of liver cancer *n* (%)			0.229
No	15 (51.7)	14 (48.3)	
Yes	0 (0.0)	3 (100.0)	
HBV infection *n* (%)			0.712
No	4 (40.0)	6 (60.0)	
Yes	11 (50.0)	11 (50.0)	
HCV infection *n* (%)			0.999
No	14 (46.7)	16 (53.3)	
Yes	1 (50.0)	1 (50.0)	
Hypertension *n* (%)			0.265
No	12 (54.5)	10 (45.5)	
Yes	3 (30.0)	7 (70.0)	
Diabetes *n* (%)			0.402
No	13 (52.0)	12 (48.0)	
Yes	2 (28.6)	5 (71.4)	
Cardiovascular and cerebrovascular diseases *n* (%)			0.589
No	13 (44.8)	16 (55.2)	
Yes	2 (66.7)	1 (33.3)	
Cirrhosis *n* (%)			0.999
No	6 (50.0)	6 (50.0)	
Yes	9 (45.0)	11 (55.0)	
Portal vein cancer thrombus *n* (%)			0.479
No	9 (56.3)	7 (43.8)	
Yes	6 (37.5)	10 (62.5)	
Number of lesions *n* (%)			**0.046**
Single lesion	0 (0.0)	5 (100.0)	
Multiple lesions	15 (55.6)	12 (44.4)	
Lymph node metastasis *n* (%)			0.589
No	13 (44.8)	16 (55.2)	
Yes	2 (66.7)	1 (33.3)	
Extrahepatic metastasis *n* (%)			0.999
No	13 (48.1)	14 (51.9)	
Yes	2 (40.0)	3 (60.0)	
CNLC stage *n* (%)			0.699

Ib~ IIb	5 (55.6)	4 (44.4)	
IIIa~ IIIb	10 (43.5)	13 (56.5)	
AJCC stage *n* (%)			0.999
II~ IIIA	5 (55.6)	4 (44.4)	
IIIB~ IVB	11 (47.8)	12 (52.2)	
Serplulimab/Bevacizumab+TACE/HAIC cycles *n* (%)			0.038
≥3 cycles	11 (39.3)	17 (60.7)	
＜3 cycles	4 (100.0)	0 (0.0)	

n: number; CNLC: China Liver Cancer Staging System; AJCC: American Joint Committee on Cancer; HBV: Hepatitis B Virus; HCV: Hepatitis C Virus; TACE: Transarterial Chemoembolization; HAIC: Hepatic Arterial Infusion Chemotherapy. Fisher's exact test.
