# Supplementary material for: Hepatic Arterial Infusion Chemotherapy with Serplulimab and the Bevacizumab Biosimilar HLX04 for Advanced Hepatocellular Carcinoma: A Prospective, Observational Phase II Clinical Trial
Source: Cancers (Basel). 2025 Oct 5;17(19):3235. doi: 10.3390/cancers17193235 (PMC12523560; doi:10.3390/cancers17193235)
Supplement: Supplementary file 1 [file cancers-17-03235-s001.zip › Supplementary Table S2.pdf]

Supplementary Table S2. Univariate Cox proportional hazards regression analysis for PFS

Index	HR	95%CI of HR		*P* value
		Lower	Upper	
Age (>60 years vs. ≤60 years)	0.527	0.118	2.364	0.403
Sex (Male vs. Female)	0.570	0.110	2.952	0.503
Drinking history (Yes vs. No)	1.588	0.354	7.116	0.546
Smoking history (Yes vs. No)	6.223	0.748	51.804	0.091
Family history of liver cancer (Yes vs. No)	1.958	0.234	16.366	0.535
HBV infection (Yes vs. No)	1.203	0.232	6.224	0.826
HCV infection (Yes vs. No)	0.042	0.000	2715.338	0.575
Hypertension (Yes vs. No)	1.674	0.372	7.541	0.502
Diabetes (Yes vs. No)	0.669	0.080	5.587	0.711
Cardiovascular and cerebrovascular diseases (Yes vs. No)	0.044	0.000	19917.290	0.638
Cirrhosis (Yes vs. No)	0.895	0.199	4.019	0.884
Portal vein cancer thrombus (Yes vs. No)	0.455	0.088	2.353	0.348
Number of lesions (Multiple vs. Single)	24.880	0.001	480446.507	0.523
Lymph node metastasis (Yes vs. No)	0.042	0.000	1683.070	0.557
Extrahepatic metastasis (Yes vs. No)	0.823	0.099	6.860	0.857
CNLC stage (IIIa~ IIIb vs. Ib~ IIb)	0.488	0.109	2.189	0.349
AJCC stage (IIIB~ IVB vs. II~ IIIA)	0.918	0.178	4.746	0.919
Serplulimab/Bevacizumab+TACE/HAIC cycles (≥3 cycles vs. <3 cycles)	0.541	0.064	4.545	0.572

CNLC: China Liver Cancer Staging System; AJCC: American Joint Committee on Cancer; HBV: Hepatitis B Virus; HCV: Hepatitis C Virus; TACE: Transarterial Chemoembolization; HAIC: Hepatic Arterial Infusion Chemotherapy.
