# Supplementary material for: Hepatic Arterial Infusion Chemotherapy with Serplulimab and the Bevacizumab Biosimilar HLX04 for Advanced Hepatocellular Carcinoma: A Prospective, Observational Phase II Clinical Trial
Source: Cancers (Basel). 2025 Oct 5;17(19):3235. doi: 10.3390/cancers17193235 (PMC12523560; doi:10.3390/cancers17193235)
Supplement: Supplementary file 1 [file cancers-17-03235-s001.zip › Supplementary Table S3.pdf]

Supplementary Table 3. AEs of the study.

AE	Any AE	Grade I	Grade II	Grade III	Grade IV
Overall Any AE	24 (75.0)	2 (6.2)	6 (18.8)	12 (37.5)	4 (12.5)
GGT increased	18 (56.2)	5 (15.6)	7 (21.9)	6 (18.8)	0 (0.0)
Hypoalbuminemia	18 (56.2)	12 (37.5)	6 (18.8)	0 (0.0)	0 (0.0)
Lymphocyte count decreased	18 (56.2)	5 (15.6)	7 (21.9)	6 (18.8)	0 (0.0)
ALP increased	18 (56.2)	13 (40.6)	3 (9.4)	2 (6.2)	0 (0.0)
AST increased	16 (50.0)	9 (28.1)	2 (6.2)	4 (12.5)	1 (3.1)
Anemia	16 (50.0)	10 (31.2)	5 (15.6)	1 (3.1)	0 (0.0)
Platelet count decreased	14 (43.8)	7 (21.9)	2 (6.2)	5 (15.6)	0 (0.0)
Bilirubin increased	14 (43.8)	3 (9.4)	6 (18.8)	4 (12.5)	1 (3.1)
White blood cell count decreased	13 (40.6)	5 (15.6)	5 (15.6)	3 (9.4)	0 (0.0)
Neutrophil count decreased	12 (37.5)	2 (6.2)	6 (18.8)	4 (12.5)	0 (0.0)
LDH increased	11 (34.4)	11 (34.4)	0 (0.0)	0 (0.0)	0 (0.0)
Hyperglycemia	11 (34.4)	11 (34.4)	0 (0.0)	0 (0.0)	0 (0.0)
Hyponatremia	10 (31.2)	8 (25.0)	0 (0.0)	1 (3.1)	1 (3.1)
ALT increased	9 (28.1)	7 (21.9)	0 (0.0)	2 (6.2)	0 (0.0)
Hypocalcemia	7 (21.9)	4 (12.5)	3 (9.4)	0 (0.0)	0 (0.0)
Hypokalemia	5 (15.6)	3 (9.4)	0 (0.0)	2 (6.2)	0 (0.0)
Hypomagnesemia	4 (12.5)	4 (12.5)	0 (0.0)	0 (0.0)	0 (0.0)
Creatinine increased	3 (9.4)	3 (9.4)	0 (0.0)	0 (0.0)	0 (0.0)
Hyperuricemia	3 (9.4)	3 (9.4)	0 (0.0)	0 (0.0)	0 (0.0)
Hypercalcemia	3 (9.4)	3 (9.4)	0 (0.0)	0 (0.0)	0 (0.0)
INR increased	2 (6.2)	1 (3.1)	1 (3.1)	0 (0.0)	0 (0.0)
Troponin I increased	2 (6.2)	2 (6.2)	0 (0.0)	0 (0.0)	0 (0.0)
APTT prolonged	2 (6.2)	2 (6.2)	0 (0.0)	0 (0.0)	0 (0.0)
Eosinophilia	1 (3.1)	1 (3.1)	0 (0.0)	0 (0.0)	0 (0.0)
Hypertriglyceridemia	1 (3.1)	1 (3.1)	0 (0.0)	0 (0.0)	0 (0.0)
Hypercholesterolemia	1 (3.1)	0 (0.0)	0 (0.0)	0 (0.0)	1 (3.1)
Hyperkalemia	1 (3.1)	1 (3.1)	0 (0.0)	0 (0.0)	0 (0.0)
Hypermagnesemia	1 (3.1)	1 (3.1)	0 (0.0)	0 (0.0)	0 (0.0)

GGT: Gamma-glutamyl transferase; ALP: Alkaline phosphatase; AST: Aspartate aminotransferase; ALT: Alanine aminotransferase; LDH: Lactate dehydrogenase.
