# Supplementary figures and images for: Hepatic Arterial Infusion Chemotherapy with Serplulimab and the Bevacizumab Biosimilar HLX04 for Advanced Hepatocellular Carcinoma: A Prospective, Observational Phase II Clinical Trial
Source: Cancers (Basel). 2025 Oct 5;17(19):3235. doi: 10.3390/cancers17193235 (PMC12523560; doi:10.3390/cancers17193235)

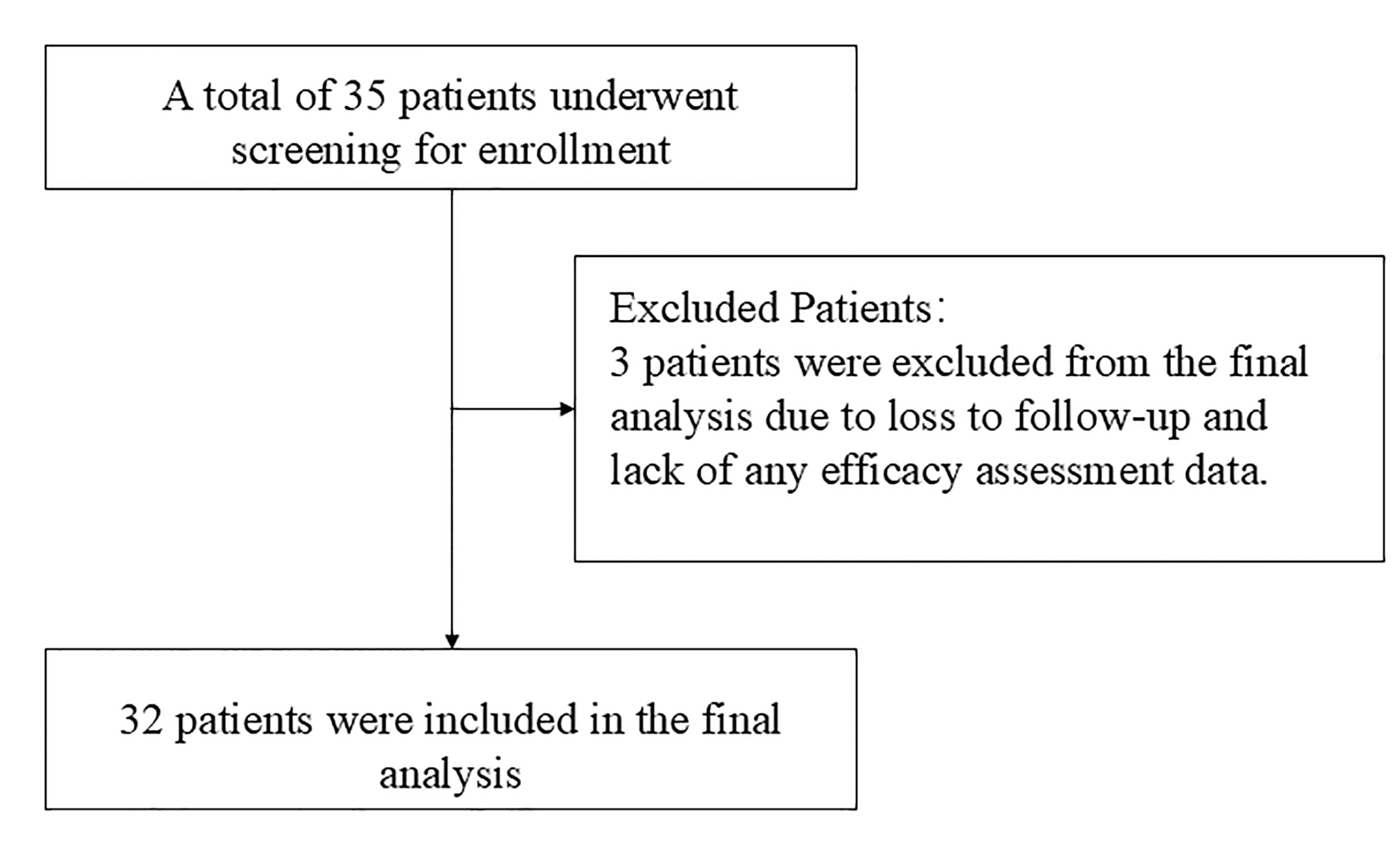

Supplement: Supplementary file 1 [file cancers-17-03235-s001.zip › Supplementary Figure S1.tif]
